# Proceedings of the Rock River Union Medical Society

**Published:** 1860-08

**Authors:** G. W. Phillips

**Affiliations:** Secretary


### PROCEEDINGS OF THE ROCK RIVER UNION MEDICAL SOCIETY.

The Annual Meeting of the Society, was held at Morrison, Whitesides Co., June 13th and 14th.

The members convened at Masonic Hall. The President, Dr. H. C. Donaldson, having called the meeting to order, the proceedings of the last meeting were read by the Secretary, and on motion accepted. Constitution read.

Drs. A. G. Porter, H. M. Freas, Arthur Nowlan, and E. M. Winters, being proposed as members of Society, were on motion admitted.

The reading of the President's address being called for, Dr. Donaldson stated that, in place of an address, he had prepared an essay; subject—" Delivery of the Placenta"—which he proceeded to read. Dr. Donaldson advocated the earlier introduction of the hand into the uterus, in case of retained Placenta, for its removal, than he believed to have been usually practiced; that the danger of introducing the hand into the uterus, had been exaggerated; that in cases of retained Placenta, the introduction of the hand is not only the best means for removing the Placenta, but is generally the only means of forming a correct diagnosis.

On motion of Dr. Hudson, the essay was accepted, and the Secretary directed to place it among the society's papers.

Dr. Hudson made some remarks "on the appearance of the tongue in disease—its value as a diagnostic sign."

Dr. H. believed the condition of the tongue by itself an uncertain guide generally; the fur or coat upon the tongue, is not of itself indicative of any particular condition, it is not a good reason to give a purgative, because the tongue is coated.

Drs. Coe and Porter believed the condition of the tongue to be an important guide, when grouped with other symptoms.

A discussion sprung up in renard to the abortive or cutting-short treatment of disease, which was participated in by Drs. Coe, Winters, Porter and Hudson.

Dr. Phillips reported two cases of amputation of the thigh, the limb having become gangrenous on account of a severe injury; in both cases the gangrene was extending at time of amputation; in one case the recovery was rapid: in the other the patient died the fourth day after amputation, from the effect of purulent absorption, or some product absorbed from the decomposing limb, an erysipelatous inflammation, attacking simultaneously, the stump, face and lungs.

On motion, adjourned until evening session.

Dr. Coe related a case of calculus of bladder, a fistulous opening extending from a point near the crest of right ilium to the bladder, through which the stone could be felt by a probe, and urine passed at times. The patient dying, the post-mortem revealed a large stone in the bladder; left ureter thickened and enlarged, the left kidney was a mere sac, the organ having been broken down and absorbed, leaving only the fibrous covering or capsule.

On motion of Dr. Hudson, a committee of two was appointed by the President, consisting of Drs. Hudson and Utley, to collect facts in relation to the recent tornado and report to the Secretary of the Smithsonian Institute.

The Society proceeded to the election of officers for the ensuing year, with the following result:

President—Dr. A. G. Porter.

Vice President—Dr. H. M. Freas.

Secretary—Dr. G. W. Phillips.

Treasurer—Dr. Moses M. Royer.

Dr. Porter upon taking the chair made a few pertinent remarks, thanking the Society for the honor conferred.

On motion, three censors were appointed, consisting of Drs. Donaldson, Royer and Freas.

On motion the Secretary was instructed to draft a memorial to be signed by the officers of the Society praying the Legislature to pass a law for the registration of births, marriages, and deaths.

Committee upon fee-bills reported in full, report adopted, and Secretary instructed to ascertain previous to next meeting of the Society, the expense of publishing 100 copies of the same.

Adjourned until after public address.

The Society having convened in the Public Hall, Dr. A. S. Hudson being introduced to the audience, gave as the subject of address, "Light, or the Sunbeam," the delivery of which was listened to with fixed attention.

The Society having convened at Masonic Hall, on motion of Dr. Hudson, Dr. A. G. Porter was chosen to deliver the address at next meeting.

On motion of Dr. Hudson, Dixon was selected as the place for holding next meeting.

On motion of Dr. Donaldson, the Secretary was instructed to furnish a copy of the proceedings for publication in the *Chicago Medical Journal.*

Discussion upon delivery of placenta by Phillips, Donaldson, Utley, Coe, and Hudson. Many of the members stated their practice to be not to wait (after having severed the cord and disposed of the child,) for a return of pain as directed by most authors, but to proceed at once to the delivery of the placenta, excititng pain by traction upon cord, and grasping the uterus through the walls of the abdomen; alleging that the sooner the placenta is expelled the less danger there will be of accident as flooding, hour-glass contraction &c.

Dr. Utley read from his note book a description of cases of diphtheria occurring at Como last winter; false membrane first appearing upon the fauces, then extending to the larynx, consequently death by suffocation; mortality small; no fever attending the affection; pulse soft; convalescence slow. Dr. Utley believes cold water applied to the throat at the commencement of the disease the sheet anchor; if this fail, next best remedies, mercurials and chlorate of potash.

Dr. Phillips related a case of diphtheria occurring at Dixon last winter, the patient a little boy three years old; the false membrane did not form upon the fauces, but formed primarily within the larynx and trachea and was expectorated as a dense, pearly white membrane, in a violent paroxysm of coughing and suffocation; a partial formation of the membrane occurred twice after this, but was dislodged by emetics; no fever in this case; pulse weak; tongue covered with a thin, moist, yellowish fur, dry and smooth at tip the third and fourth days; patient recovered; treatment, calomel in two gr. doses every two hours, continued right along until gums were affected, a few free doses of sulph., quinea, nitrate of silver to fauces, and emetics.

On motion, four committees were appointed by the President consisting of three members on each committee, one upon surgery, consisting of Drs. Utley, Phillips, and W. W. Winters: upon practical medicine, consisting of Drs. Freas, Donaldson, and Geo. W. Hewitt; upon obstetrics, consisting of Drs. Hudson, Donaldson, and Edward Winters; and one upon the best means of preparing medicines for convenient administration to young children, composed of Drs. Coe, Abbott, and Jackson.

On motion, the Society adjourned.

G. W. PHILLIPS, M. D.,
*Secretary.*

